# Molecular characterization of *G6PD* mutations identifies new mutations and a high frequency of intronic variants in Thai females

**DOI:** 10.1371/journal.pone.0294200

**Published:** 2023-11-15

**Authors:** Kamonwan Chamchoy, Sirapapha Sudsumrit, Jutamas Wongwigkan, Songsak Petmitr, Duantida Songdej, Emily R. Adams, Thomas Edwards, Ubolsree Leartsakulpanich, Usa Boonyuen

**Affiliations:** 1 Princess Srisavangavadhana College of Medicine, Chulabhorn Royal Academy, Bangkok, Thailand; 2 Department of Molecular Tropical Medicine and Genetics, Faculty of Tropical Medicine, Mahidol University, Bangkok, Thailand; 3 Department of Pediatrics, Faculty of Medicine Ramathibodi Hospital, Mahidol University, Bangkok, Thailand; 4 Centre for Drugs and Diagnostics Research, Liverpool School of Tropical Medicine, Liverpool, United Kingdom; 5 National Center for Genetic Engineering and Biotechnology, National Science and Technology Development Agency, Pathum Thani, Thailand; Shoklo Malaria Research Unit, THAILAND

## Abstract

Glucose-6-phosphate dehydrogenase (G6PD) deficiency is an X-linked enzymopathy caused by mutations in the *G6PD* gene. A medical concern associated with G6PD deficiency is acute hemolytic anemia induced by certain foods, drugs, and infections. Although phenotypic tests can correctly identify hemizygous males, as well as homozygous and compound heterozygous females, heterozygous females with a wide range of G6PD activity may be misclassified as normal. This study aimed to develop multiplex high-resolution melting (HRM) analyses to enable the accurate detection of *G6PD* mutations, especially among females with heterozygous deficiency. Multiplex HRM assays were developed to detect six *G6PD* variants, i.e., *G6PD* Gaohe (c.95A>G), *G6PD* Chinese-4 (c.392G>T), *G6PD* Mahidol (c.487G>A), *G6PD* Viangchan (c.871G>A), *G6PD* Chinese-5 (c.1024C>T), and *G6PD* Union (c.1360C>T) in two reactions. The assays were validated and then applied to genotype *G6PD* mutations in 248 Thai females. The sensitivity of the HRM assays developed was 100% [95% confidence interval (CI): 94.40%–100%] with a specificity of 100% (95% CI: 88.78%–100%) for detecting these six mutations. The prevalence of G6PD deficiency was estimated as 3.63% (9/248) for G6PD deficiency and 31.05% (77/248) for intermediate deficiency by phenotypic assay. The developed HRM assays identified three participants with normal enzyme activity as heterozygous for *G6PD* Viangchan. Interestingly, a deletion in intron 5 nucleotide position 637/638 (c.486-34delT) was also detected by the developed HRM assays. *G6PD* genotyping revealed a total of 12 *G6PD* genotypes, with a high prevalence of intronic variants. Our results suggested that HRM analysis-based genotyping is a simple and reliable approach for detecting *G6PD* mutations, and could be used to prevent the misdiagnosis of heterozygous females by phenotypic assay. This study also sheds light on the possibility of overlooking intronic variants, which could affect *G6PD* expression and contribute to enzyme deficiency.

## Introduction

Glucose-6-phosphate dehydrogenase (G6PD) is an enzyme that catalyzes the production of reduced nicotinamide adenine dinucleotide phosphate (NADPH), a reducing agent that plays an important role in protecting cells against oxidative stress. G6PD is especially critical in red blood cells because it is the only source of NADPH [1]. G6PD deficiency is an X-linked genetic disorder caused by mutations in the *G6PD* gene. It affects approximately 500 million people worldwide, mostly among populations living in malaria-endemic (or formerly endemic) areas [2]. The distribution of G6PD deficiency across malaria-endemic areas was postulated to be associated with the natural selection by survival advantage against malaria infection [2, 3]. The human *G6PD* gene consists of 13 exons and 12 introns, among which the first exon is an untranslated region (UTR) and the start codon ATG is located at the 5’ end of exon 2 [4]. As an X-linked disorder, the clinical manifestations of G6PD deficiency are more prominent in hemizygous males, as well as homozygous and compound heterozygous females. Females who are heterozygous for G6PD deficiency exhibit a wide range of enzyme activity because they have mixed populations of G6PD-normal and G6PD-deficient red blood cells as a result of random X-chromosome inactivation (or lyonization) during embryonic development [5].

Individuals with G6PD deficiency are mostly asymptomatic. The clinical manifestations emerge when red blood cells are exposed to agents causing oxidative stress, such as compounds in fava beans, drugs, or infections. Acute hemolytic anemia is the most common manifestation of G6PD deficiency. The severity of hemolysis can vary from mild to severe and life-threatening, depending on the level of oxidative stress and the degree of enzymatic dysfunction. Acute hemolytic crisis in individuals with deficient or intermediate G6PD activity is a medical problem associated with the use of primaquine and tafenoquine, the only two 8-aminoquinoline drugs approved for the radical treatment of malaria [6–8]. Primaquine and tafenoquine are indicated to prevent relapse by killing liver-stage *Plasmodium vivax* and *P*. *ovale*. However, these 8-aminoquinolines can cause hemolytic toxicity in G6PD deficient as well as intermediate patients [6–8]. In particular, concerns have been raised regarding hemolytic risk in heterozygous females with G6PD activity comparable to that of normal individuals [7]. Not only 8-aminoquinolines, but also other drugs, have been demonstrated to trigger acute hemolysis in G6PD-deficient individuals [9–11]. Furthermore, in neonates, G6PD deficiency is associated with hyperbilirubinemia, leading to severe kernicterus [2].

To date, over 200 *G6PD* variants have been identified and reported worldwide. Approximately 85% of reported *G6PD* variants involve exonic mutations, causing amino acid change and altering enzyme properties [12]. In Thailand, the prevalence of G6PD deficiency ranges between 7% and 20%, depending on the geographical area and ethnicity. More than 20 *G6PD* variants have been identified, with *G6PD* Mahidol (c.487G>A, Class B) and *G6PD* Viangchan (c.871G>A, Class B) being predominant [13–21]. The diagnosis of G6PD deficiency is not routinely practiced in Thailand; it is usually performed for patients with symptoms of unexplained hemolytic anemia. However, according to previous studies, G6PD deficiency is highly prevalent in Thailand, particularly among populations residing in malaria-endemic areas, and Class B variants that trigger hemolysis have been found to be common [13, 14, 19]. G6PD status is important health information for avoiding certain foods and chemicals that can cause hemolytic toxicity. Therefore, it would be appropriate to determine G6PD status before prescribing oxidation-inducing drugs. A family history of G6PD deficiency is also useful for predicting the risk of hyperbilirubinemia in newborns.

G6PD deficiency can be detected by various means, including phenotypic and genotypic tests. Several methods for conducting phenotypic assays are available, such as the fluorescence spot test (FST) and rapid diagnostic tests, but these are qualitative methods that can accurately detect complete deficiency where enzyme activity is less than 30% of normal [22, 23]. It should be noted that a hemizygote for the *G6PD* Mahidol variant was misdiagnosed as normal by FST, and the patient was prescribed a primaquine regimen, which resulted in severe hemolysis and coma [6]. Meanwhile, a spectrophotometric method, where enzyme activity is measured at 340 nm, is available as a quantitative assay. Despite being the gold standard, access to spectrophotometric determination is limited in malaria-endemic regions where G6PD deficiency is common and critical, because the technique requires laboratory competence and equipment [24]. In addition, spectrophotometry is sometimes unable to identify heterozygous females who have normal G6PD activity but are still susceptible to drug-induced hemolysis when exposed to oxidative agents [7]. Hence, alternative approaches, such as genetic analysis, could be useful to determine whether drugs should be administered to people with suspected G6PD deficiency. In addition, *G6PD* genotyping overcomes the possibility of a false negative/positive diagnosis in quantitative methods when a change in G6PD activity occurs due to various hematological factors. *G6PD* genotyping can be performed based on methods such as restriction fragment length polymorphism (RFLP), amplification-refractory mutation system (ARMS), and sequencing. However, these methods are time-consuming, as many steps are required to complete the genotyping process [25, 26]. High-resolution melting (HRM) analysis is a rapid and reliable tool for detecting *G6PD* mutations. This assay successfully distinguishes the zygosity of *G6PD* variants. However, previous HRM assays could detect only one or two mutations in a single reaction [27–29].

The aim of this study is to develop multiplex HRM assays that enable a high-throughput platform for detecting *G6PD* mutations and identifying zygosity, and to determine the prevalence and molecular characteristics of G6PD deficiency among Thai females.

## Materials and methods

### Blood samples and DNA extraction

This retrospective study was carried out using archived blood samples. The data were fully anonymized and the authors had no access to information that could identify individual participants.

The development and validation of multiplex HRM assays were performed using blood samples collected from Thai volunteers at the Faculty of Medicine Ramathibodi Hospital, Bangkok, Thailand in 2019–2020. All samples used in developing and validating the method were previously genotyped by Sanger sequencing.

For G6PD characterization, blood samples collected from Thai females at the Hospital for Tropical Diseases, Bangkok, Thailand, in 2015, were used. The minimum sample size was calculated based on the prevalence of G6PD deficiency among females previously reported at a hospital in central Thailand [18], using the following equation: $n=\frac{Z^{z}PQ}{D^{2}}$,

where z = critical value = 1.96;

P = prevalence of G6PD deficiency by genotyping = 12.5% = 0.125;

Q = 1‐P = 0.875;

D = allowance for error = 5% = 0.05.

A sample size of 248 females was thereby sufficient for this study. Blood samples from 248 Thai females were genotyped for *G6PD* using multiplex HRM assays and phenotypically determined for their G6PD enzyme activity. To maintain the integrity of samples for phenotypic analysis, blood samples were collected in ethylenediaminetetraacetic acid (EDTA) tubes, aliquoted, and stored at −20°C until use [30].

Genomic DNA extraction was performed using a QIAamp DNA Blood Mini Kit (QIAGEN, Hilden, Germany), according to the manufacturer’s instructions. Blood samples of 200 μL were extracted and eluted into a final volume of 100 μL. DNA concentration was measured using a NanoDrop 2000 spectrophotometer (Thermo Fisher Scientific, Waltham, MA, USA).

### Development of multiplex HRM assays for identifying heterozygous G6PD deficiency

**Fig 1:**
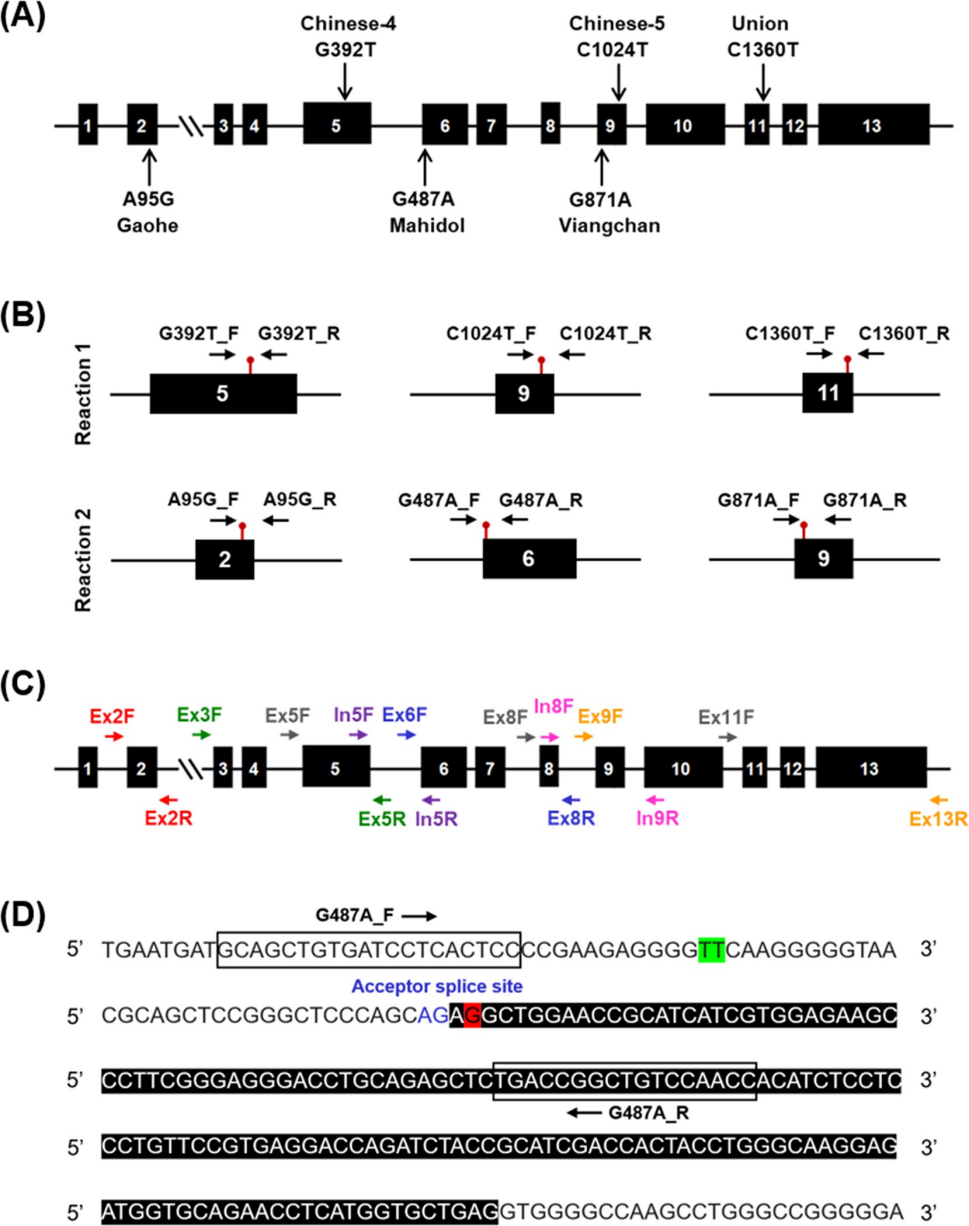
Map of *G6PD* gene and positions of primers used in this study. Exons are shown in black boxes. The primers are illustrated with arrows. (A) Distribution of six mutations along the exon coding sequence. (B) Primer schematic for HRM analysis. Point mutations are indicated by red pins. (C) Primer schematic for *G6PD* direct sequencing. (D) Location of HRM primers for detecting *G6PD* Mahidol. Exon 6 is shaded in black. Nucleotide position 637/638 of intron 5 and *G6PD* Mahidol mutation are highlighted in green and red, respectively. Boxes indicate primer binding sites. Blue text indicates the acceptor splice site of intron 5.

Multiplex HRM assays were developed to detect six *G6PD* variants previously reported in Thailand, namely, *G6PD* Gaohe (c.95A>G), *G6PD* Chinese-4 (c.392G>T), *G6PD* Mahidol (c.487G>A), *G6PD* Viangchan (c.871G>A), *G6PD* Chinese-5 (c.1024C>T), and *G6PD* Union (c.1360C>T), Fig 1A. We expanded previously established HRM assays and used multiplex analysis to simultaneously detect three variants in a single reaction [27]. Primers were designed flanking each target point mutation in the *G6PD* gene (Fig 1B), to generate amplicons with different melting temperatures (*T*_*m*_) depending on the presence/absence of the mutation (Table 1). The theoretical *T*_*m*_ of PCR products was predicted by the OligoCalc online software (http://biotools.nubic.northwestern.edu/Oligoalc.html) using the nearest neighbor calculation.

**Table 1:** Primers used in multiplex HRM assays.

Reaction	*G6PD* variant	Primer name	Primer sequence (5’ to 3’)	Primer concentration (nM)	Amplicon size (bp)
1	Chinese-4	G392T_F^a^	TACCAGCGCCTCAACAGC	200	75
		G392T_R	CAAGGCCAGGTAGAAGAG		
	Chinese-5	C1024T_F^a^	CACTTTTGCAGCCGTCGT	300	65
		C1024T_R^a^	CTCGAAGGCATCACCTACCA		
	Union	C1360T_F	CTGCGGGAGCCAGATGCACT	200	104
		C1360T_R	GTGGCACACAGGGAGGGA		
2	Gaohe	A95G_F^a^	GGCGATGCCTTCCATCAGTC	300	109
		A95G_R^a^	AGGCATGGAGCAGGCACTTC		
	Mahidol	G487A_F^a^	GCAGCTCTGATCCTCACTCC	200	137
		G487A_R^a^	GGTTGGACAGCCGGTCA		
	Viangchan	G871A_F	CCCTTGGCTTTCTCTCAGGTC	300	55
		G871A_R^a^	TGGCCTGCACCTCTGAGAT		

^a^ Primers were previously reported by Yan, et al., 2010 [27].

A reaction volume of 12.5 μL was used for the multiplex HRM assays. The reaction mixture contained 6.25 μL of 2× HRM Type-It mix (QIAGEN), various concentrations of primers (Table 1), molecular-grade water, and 2.5 μL of the gDNA template (15–20 ng/μL). PCR amplification and melting-curve analysis were performed on a Rotor-Gene Q real-time instrument (QIAGEN). The amplification conditions were 1 cycle of 95°C for 5 min, followed by 30 cycles of 95°C for 5 s, 63°C for 30 s, and 72°C for 10 s. Subsequently, a melting-curve profile was generated by increasing the temperature from 70°C to 90°C with an increment of 0.1°C per 2 s, with data acquired in the HRM channel. DNA samples with known *G6PD* genotypes (confirmed by Sanger sequencing) were included in every run as wild-type and positive (mutant) controls. Data analysis was conducted using Rotor-Gene Q software.

### Validation of multiplex HRM assays

To assess the performance of the developed HRM assays for detecting six *G6PD* variants, the number of true positives (TP), true negatives (TN), false positives (FP), and false negatives (FN) were determined using Sanger sequencing as a reference method. A total of 95 blood samples with known *G6PD* genotypes (64 *G6PD*-mutant and 31 wild-type, S1 Table) were used to determine the specificity and sensitivity of the assay. Sensitivity is the proportion of mutant samples correctly identified for its zygosity, while specificity is the proportion of wild-type samples correctly identified as negative [31, 32].

### Phenotypic and genotypic characterization of G6PD deficiency among Thai females

This study aimed to develop genotypic assays for identifying heterozygous G6PD deficiency. A total of 248 blood samples from Thai females were used. G6PD phenotyping was carried out using WST-8 assay. A whole-blood sample was mixed with a reaction mixture containing 20 mM Tris-HCl pH 8.0, 10 mM MgCl_2_, 500 μM glucose-6-phosphate (G6P), 100 μM NADP^+^, and 100 μM WST-8 (Sigma-Aldrich, Darmstadt, Germany) in a 96-well plate. The enzymatic reaction was followed at 450 nm using a microplate reader (Sunrise; Tecan, Männedorf, Switzerland). Absorbance at 450 nm of a reaction mixture set up in the absence of substrates was used for background subtraction. Hemoglobin concentration was measured using Drabkin’s reagent (Sigma-Aldrich). G6PD activity was reported as units (U) per gram of hemoglobin (gHb).

**Fig 2:**
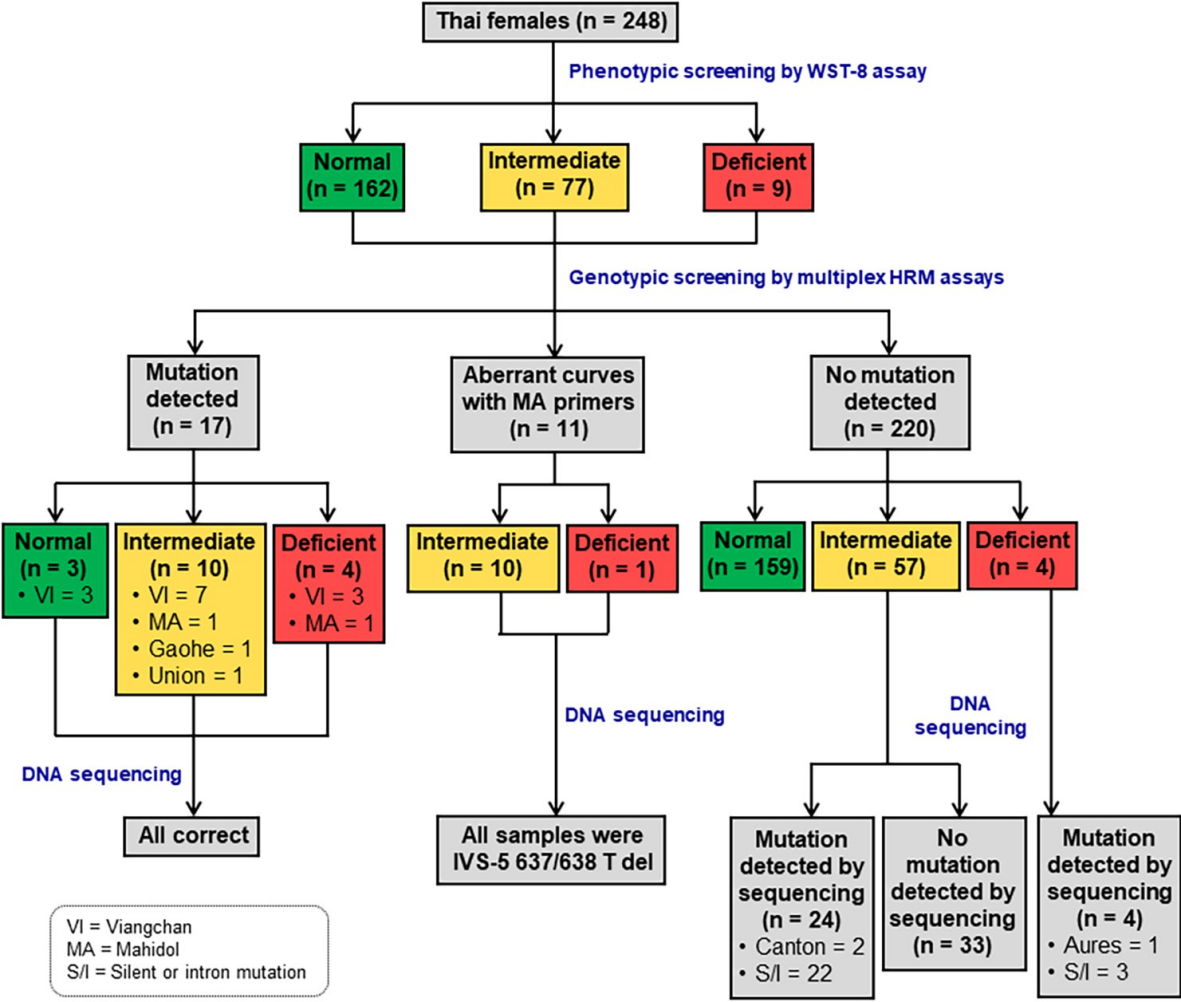
Flow chart of study procedure.

*G6PD* genotyping was performed with 248 samples using the developed HRM assays, Table 1, according to the protocols outlined above. The study procedure is depicted in Fig 2.

### PCR amplification and Sanger sequencing

Following a previous report with modifications, the coding sequence of *G6PD* gene (exons 2 to 13) was amplified (Fig 1C and S2 Table) [14]. The PCR reaction was set up in a total volume of 50 μL, consisting of 1× Taq Buffer with (NH_4_)_2_SO_4_, 2.5 mM MgCl_2_, 200 μM of each dNTP, 0.25 μM of each primer, 50 ng of gDNA, and 1.25 U of Taq DNA polymerase (Thermo Fisher Scientific). The thermal cycling profile was as follows: initial denaturation at 95°C for 3 min; 35 cycles of denaturation at 95°C for 30 s, annealing for 30 s, and extension at 72°C for 1 min; followed by final extension at 72°C for 10 min. PCR products were subjected to purification and sequenced (1st BASE; Apical Scientific, Selangor, Malaysia). DNA sequencing provided coverage for exons 2 to 13 and introns 3 to 12.

### Statistical analysis

The calculations of sensitivity and specificity were performed using MedCalc online software (https://www.medcalc.org/calc/diagnostic_test.php), according to the following parameters: sensitivity = TP/(TP+FN) ×100; specificity = TN/(TN+FP) ×100. The results were expressed as percentage with 95% confidence interval (CI). The G6PD activity of the population was expressed as median ± interquartile range (IQR) using GraphPad Prism (GraphPad Software, La Jolla, CA, USA).

## Results

### Development and validation of HRM assays

**Fig 3:**
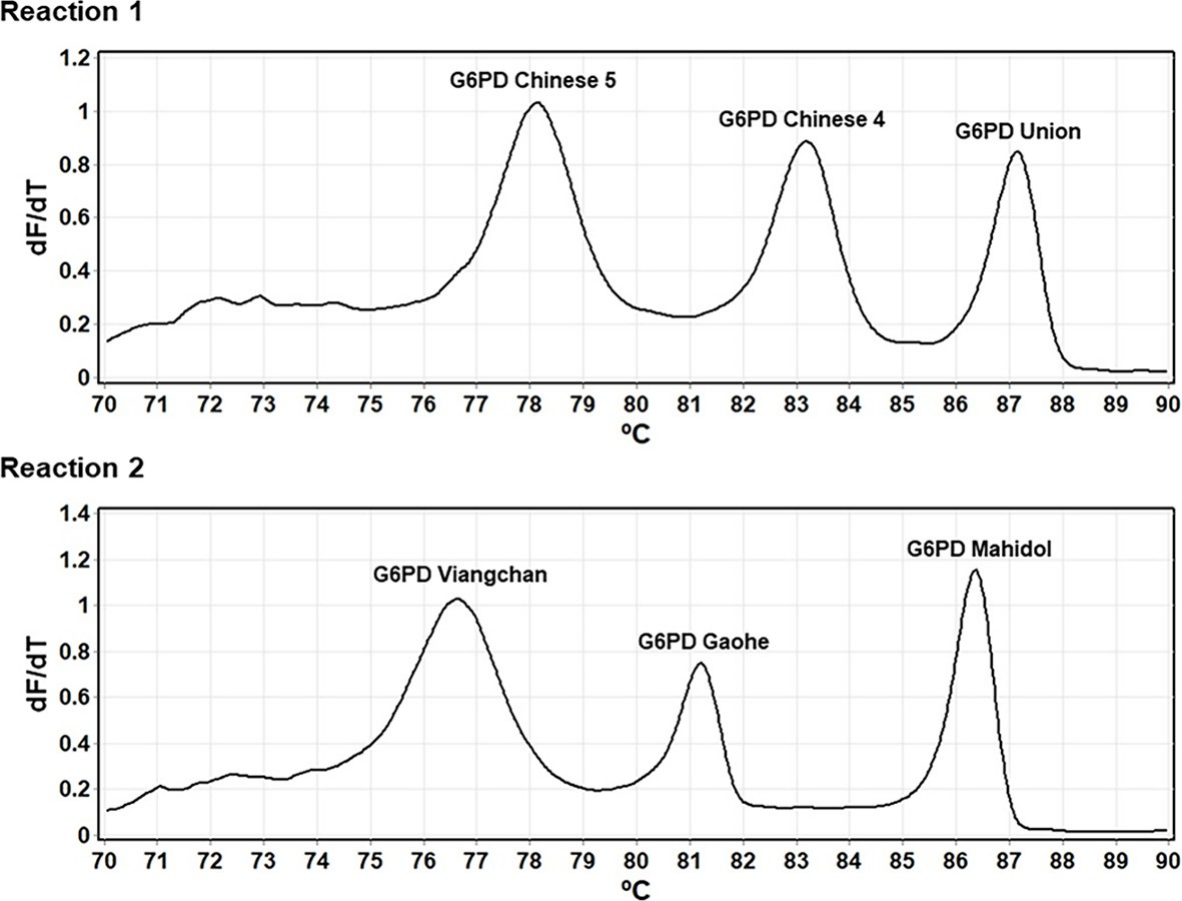
Identification of *G6PD* mutations by multiplex HRM assay. Each *G6PD* variant produces a peak at the corresponding *T*_*m*_.

**Fig 4:**
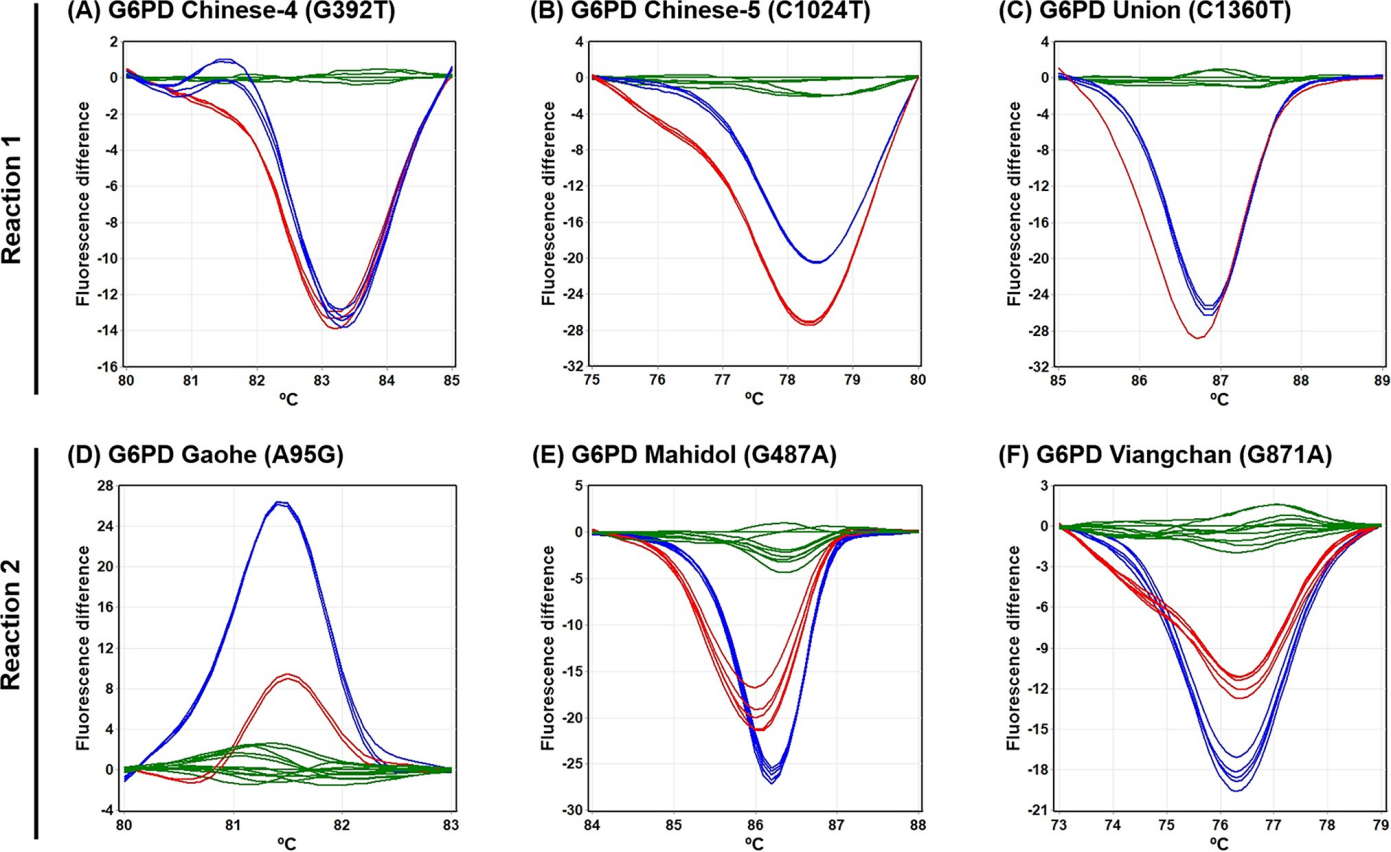
HRM curves of six *G6PD* mutations in difference plot analyses. (A) *G6PD* Chinese-4, (B) *G6PD* Chinese-5, (C) *G6PD* Union, (D) *G6PD* Gaohe, (E) *G6PD* Mahidol, and (F) *G6PD* Viangchan. Green lines represent wild-type *G6PD*. Red lines represent heterozygous females. Dark blue lines represent hemizygous *G6PD* mutants.

Previously, single-plex HRM analysis was used to screen for *G6PD* mutations in a Han Chinese population [27]. Here, to enable high-throughput detection of *G6PD* mutations, triplex HRM assays were developed to detect six *G6PD* mutations. The assay conditions, including primer concentrations, amplification cycles, and melting protocol, were optimized to maximize the sensitivity and specificity of the detection. The melting curves of each *G6PD* variant were generated at distinct temperatures (Fig 3). In addition, the zygosity of samples was detected using the difference in the melting-curve shapes and *T*_*m*_ among wild-type, heterozygous, and hemizygous/homozygous statuses (Fig 4).

The developed HRM assays were validated with 95 samples (64 *G6PD*-mutant and 31 wild-type, confirmed by DNA sequencing, S1 Table). The HRM assays were 100% sensitive [95% confidence interval (CI): 94.40%–100%] and 100% specific (95% CI: 88.78%–100%) for detecting these six mutations. As shown in the difference plots, the HRM curves of *G6PD* Chinese-4, *G6PD* Chinese-5, *G6PD* Union, *G6PD* Gaohe, *G6PD* Mahidol, and *G6PD* Viangchan for each zygosity could be determined unambiguously (Fig 4A–4F), and were correctly identified by the automated software.

### Phenotypes of G6PD deficiency among Thai females

**Fig 5:**
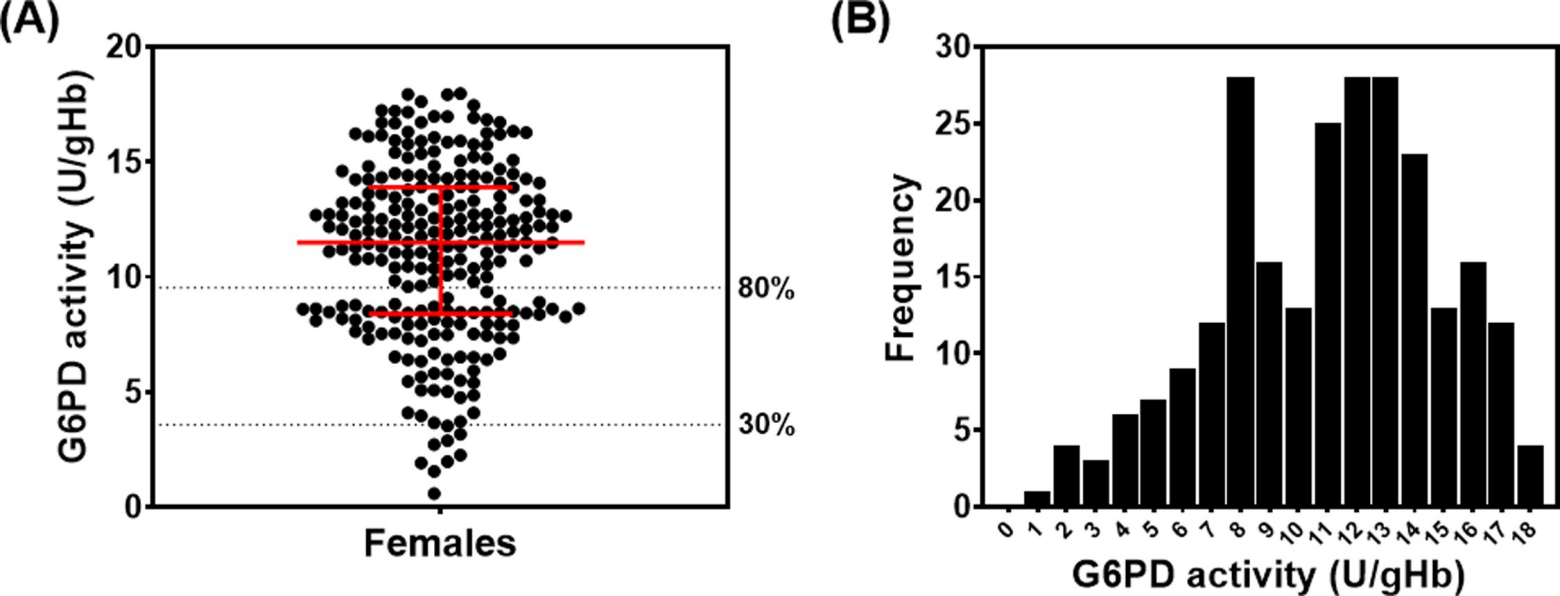
G6PD activity of 248 female individuals. (A) Distribution of G6PD activity as determined by WST-8 assay. Red lines represent median ± IQR. (B) Frequency distribution of G6PD activity in the studied population.

The G6PD enzyme activities of 248 female participants were measured using a phenotypic quantitative test, the WST-8 assay. The median enzyme activity in the studied population was 11.50 ± 5.51 U/gHb, with G6PD activity ranging from 0.59 to 17.97 U/gHb (Fig 5A). A wide range of G6PD activities was observed among the studied group, implying the presence of heterozygous G6PD deficiency (Fig 5B). Since only females were included in this study, we could not determine the adjusted male median (AMM) value among the population studied. However, from our unpublished data, the AMM was 11.93 ± 2.55 U/gHb, which was defined as 100% activity [24]. According to the World Health Organization (WHO), individuals with a G6PD activity level of < 30% of the AMM are considered to be deficient, and those with G6PD activity level between 30% and 80% of the AMM are considered intermediately deficient [33]. Therefore, 30% and 80% of the AMM (3.58 and 9.54 U/gHb) were used as thresholds for deficiency and intermediate deficiency, respectively. Overall, nine female participants (3.63%) exhibited G6PD activity <30% of the AMM and were considered G6PD-deficient. Meanwhile, 77 female participants (31.05%) who showed moderate G6PD activity, between 30% and 80% of the AMM, were identified as having intermediate deficiency (Fig 2).

### Genotypes of G6PD deficiency among Thai females

#### Identification of *G6PD* mutations by multiplex HRM assays

The developed triplex HRM assays were applied to detect six *G6PD* variants in 248 DNA samples of Thai females. *G6PD* mutations were detected in 17 samples (6.85%) of the studied population, all of which were heterozygotes. *G6PD* Viangchan was the most common variant in the studied population, accounting for 76.47% (13/17) of the total. Other variants observed by multiplex HRM assays included *G6PD* Mahidol (2 samples), *G6PD* Union (1 sample), and *G6PD* Gaohe (1 sample). Among these 17 *G6PD*-mutant samples detected by HRM assays, 3 participants (*G6PD* Viangchan) were identified as G6PD normal by phenotypic test. Ten samples had intermediate G6PD activity between 30% and 80%, and the remaining 4 samples featured G6PD deficiency with enzyme activities of <30% (Fig 2). These *G6PD*-mutant samples were subjected to DNA sequencing to confirm the mutations.

**Fig 6:**
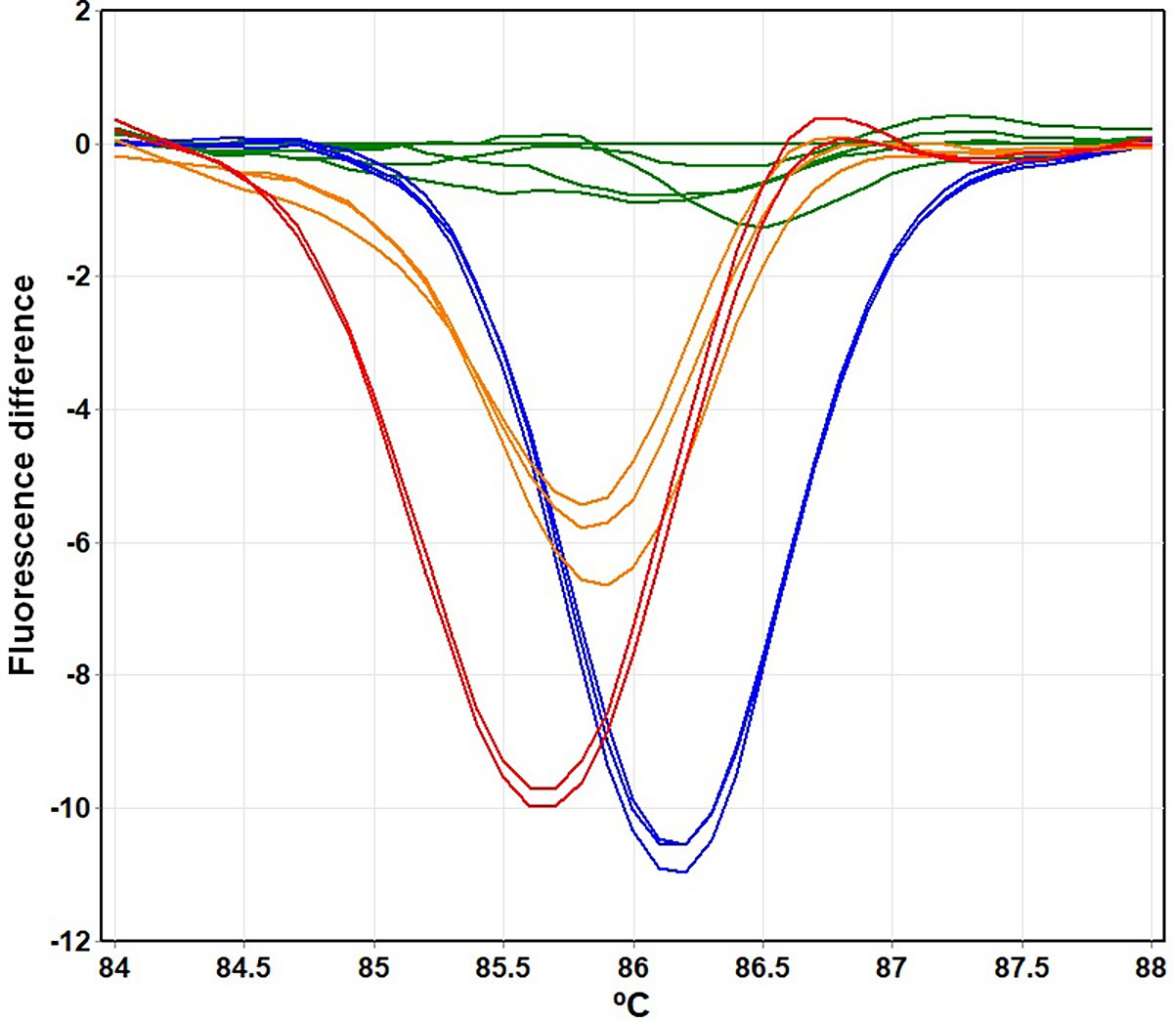
HRM curve analysis using *G6PD* Mahidol primers. Green lines represent wild-type *G6PD*. Red lines represent heterozygous females. Dark blue lines represent hemizygotes. Orange lines are aberrant melting curves.

For the remaining 72 samples with impaired G6PD activities (5 with G6PD deficiency and 67 with intermediate deficiency), no mutation was detected by HRM assays (Fig 2). Notably, among these samples, multiplex HRM assays revealed 11 samples with aberrant melting curves in the analysis using *G6PD* Mahidol primers, indicating nucleotide change within the detected amplicon (Fig 6). Hence, all of these 72 samples with impaired G6PD activity were sequenced to identify the *G6PD* mutations.

#### DNA sequencing

Among the 89 samples subjected to DNA sequencing, 56 were found to have *G6PD* mutations (Table 2). DNA sequencing confirmed the presence of *G6PD* mutation and its zygosity in all positive samples called by the HRM assays (17 samples). The sequencing results showed that 11 samples having aberrant melting curves in the analysis by *G6PD* Mahidol primers (Fig 6) had a deletion in intron 5 at position 637/638 (c.486-34delT; rs3216174). The average G6PD activity of those with the c.486-34delT was 7.11 ± 1.72 U/gHb (59.60% of the AMM), which can be defined as intermediate deficiency.

**Table 2:** *G6PD* mutations detected by DNA sequencing.

Genotype	Variant name	Effect of mutation	*N*	Frequency (%)^a^	Enzyme activity (U/gHb)^b^
**c.95A>G**	Gaohe	p.His32Arg	1	1.79	5.50
c.143T>C	Aures	p.Ile48Thr	1	1.79	1.55
**c.487G>A**	Mahidol	p.Gly163Ser	2	3.57	5.41 ± 4.94
c.519C>T		p.Phe173	1	1.79	8.14
**c.871G>A**, c.1311C>T^*^, c.1365-13T>C^*^	Viangchan	p.Val291Met, p.Tyr437, intronic variant	2	3.57	5.78 ± 2.37
**c.871G>A**, c.1311C>T, c.1365-13T>C	Viangchan	p.Val291Met, p.Tyr437, intronic variant	11	19.64	5.68 ± 3.54
c.1311C>T^*^, c.1365-13T>C^*^		p.Tyr437, intronic variant	5	8.93	6.76 ± 1.02
c.1311C>T, c.1365-13T>C		p.Tyr437, intronic variant	17	30.36	5.54 ± 1.98
**c.1360C>T**	Union	p.Arg454Cys	1	1.79	6.41
c.1376G>T	Canton	p.Arg459Leu	2	3.57	8.05 ± 0.70
c.486-34delT		Intronic frameshift mutation	11	19.64	7.11 ± 1.72
c.1365-13T>C		intronic variant	2	3.57	6.15 ± 0.51
**Total**			**56**	**100**	

^a^ Frequency was calculated as a percentage of total variants observed in the study.

^b^ G6PD activity is expressed as mean ± SD.

^*^ indicates homozygosity.

Bold indicates mutations included in HRM assays.

Additional nonsynonymous variants that were not included in the HRM assays, namely *G6PD* Canton (c.1376G>T) and *G6PD* Aures (c.143T>C), were detected. All *G6PD* mutations observed in this study are categorized as Class B variants associated with susceptibility to hemolytic toxicity. *G6PD* Canton was observed in two heterozygous females with average G6PD activity of 8.05 ± 0.70 U/gHb (67.48% of the AMM). *G6PD* Aures was found in a heterozygous participant with G6PD enzyme activity of 1.55 U/gHb (12.99% of the AMM). It was revealed that individuals carrying *G6PD* Viangchan also had two other mutations (synonymous or silent mutation c.1311C>T (rs2230037) and intron 11 mutation c.1365-13T>C; rs2071429).

Here, 25.58% (22/86) of individuals with enzyme activity <80% of the AMM value had a combination of c.1311C>T and c.1365-13T>C (17 heterozygous and 5 homozygous), in whom no other mutations were observed in the coding exons and intronic flanking regions of the *G6PD* gene. The average G6PD activity of c.1311C>T/ c.1365-13T>C was 6.76 ± 1.02 U/gHb for homozygotes and 5.54 ± 1.98 U/gHb for heterozygotes, which were approximately 50% of the normal level (Table 2). Therefore, a silent mutation of c.1311C>T in exon 11 combined with c.1365-13T>C might have resulted in impaired G6PD activity in the studied population.

DNA sequencing also revealed one sample carrying a synonymous mutation c.519C>T (Phe173). Heterozygous c.519C>T mutation was detected in a subject presenting intermediate G6PD activity (8.14 U/gHb; 68.23% of the AMM). The c.519C>T is a rare mutation that has previously been reported in Chinese, Taiwanese, and Thai populations [34–36]. The mutation can be found as a single mutation or in combination with c.1311C>T / c.1365-13T>C and 3’ UTR c.357A>G [34]. It was noted that the remaining 33 samples, which were G6PD intermediate cases, had no identifiable mutations.

## Discussion

Because individuals with deficient as well as intermediate levels of G6PD activity are at risk of developing adverse effects when receiving radical treatment for malaria, the WHO recommends G6PD testing before prescribing 8-aminoquinolines. Individuals with <30% of normal G6PD activity can receive primaquine with a dose adjustment under medical supervision [33]. On the other hand, a single dose of the long-acting tafenoquine can only be prescribed to those with >70% G6PD activity [37]. Since the prevalence of G6PD deficiency parallels malaria frequency, radical treatment by 8-aminoquinolines poses significant challenges in malaria-endemic areas, rendering diagnosis of G6PD deficiency crucial in these regions [2, 38]. Though phenotypic screening tests are potential tools for identifying G6PD deficiency, their limitation is the inability to accurately identify heterozygous females with enzyme deficiency. Growing evidence supports the integration of genetic testing for the accurate determination of G6PD deficiency status [14, 27, 39]. PCR-RFLP and the DiaPlexC G6PD Genotyping Kit are widely used for genotyping the *G6PD* gene. However, these methods require additional gel electrophoresis after PCR amplification. Furthermore, PCR-RFLP needs specific restriction enzymes for detecting each variant [25, 40]. Reverse dot blot flow-through hybridization (RDB-FTH) is a naked-eye readable assay that can simultaneously detect 14 *G6PD* variants within one run, but it also has post-PCR works. Many reagents are required for hybridization and visualization of spots on the membrane. With a manual procedure, RDB-FTH could take up to 4 h and, hence, a rapid hybridization machine is required to reduce hybridization time [41, 42]. Although DNA sequencing remains the gold standard for the detection of *G6PD* mutation, especially in female heterozygotes, this method is impractical for large-scale screening and routine laboratory testing.

In this study, we developed multiplex HRM assays for genotyping the *G6PD* gene by modifying available single-plex analyses [27]. The developed triplex HRM assays can detect *G6PD* variants and their zygosity with a run time of 90 min. Six *G6PD* mutations—*G6PD* Gaohe, *G6PD* Chinese-4, *G6PD* Mahidol, *G6PD* Viangchan, *G6PD* Chinese-5, and *G6PD* Union—were simultaneously detected in two reactions. Cost for detecting 6 mutations in one sample was estimated at approximately 2 USD, excluding PCR instrument and labor. Differences in melting-curve shapes and *T*_*m*_ can identify whether *G6PD* mutant samples are heterozygous or hemizygous/homozygous (Fig 4).

HRM assays identified three *G6PD* Viangchan samples that were phenotypically normal, with G6PD activity over the 80% cut-off. *G6PD* Viangchan is potentially associated with susceptibility to acute hemolytic anemia, which may range from mild to severe depending on residual G6PD enzyme activity [43, 44]. Interestingly, the primers that flank the *G6PD* Mahidol mutation region could detect the intronic variant, c.486-34delT (Fig 1D). The melting curves generated in samples harboring this variant deviated from those of the wild-type and the *G6PD* Mahidol variant (Fig 6).

Our study involving molecular analysis agrees well with previous studies in which *G6PD* Viangchan was found to be the most common variant in central Thailand [18, 21, 27, 45–48]. All 13 individuals carrying the c.871G>A mutation were found to have the combination of c.1311C>T in exon 11 and c.1365-13T>C in intron 11. This indicates the linkage disequilibrium between *G6PD* Viangchan and c.1311C>T/ c.1365-13T>C polymorphisms [27, 35, 49]. The combination of c.1311C>T and c.1365-13T>C were relatively frequent in the present study (Table 2). The double mutation (c.1311C>T and c.1365-13T>C) is silent, but the combination of these two polymorphisms has been found to be associated with G6PD deficiency, as it was frequently detected among individuals with reduced enzyme activity [50–53]. Nonetheless, some studies showed that the dual presence of c.1311C>T and c.1365-13T>C was common among those with normal G6PD status [27, 54]. Both c.1311C>T and c.1365-13T>C are predicted to have no splicing or deleterious effect on the *G6PD* gene [55–57]. On the ClinVar database, c.1365-13T>C was found to be benign or likely benign while its effect on pathogenicity was uncertain. The c.1311C>T variant was found to be associated with G6PD deficiency in hemizygotes although other studies suggested that it was harmless [58, 59].

An intronic variant, c.486-34delT, was found in 11 cases. This study is the first to report this deletion variant in the Thai population. The average G6PD activity in individuals carrying this variant was 7.11 ± 1.72 U/gHb (59.60% of the AMM). This deletion was previously reported as a sporadic variant in the Chinese population [54, 60–62]. In a study in Wuhan, c.486-34delT was found at a rate of 2% among *G6PD* variants, which was associated with an enzyme activity of 2.37 U/gHb [60]. Although the effect of this mutation on the expression of the *G6PD* gene has not been described, the c.486-34delT was found to be associated with reduced G6PD activity in the studied population. Because nucleotide position 637/638 is located approximately 630 and 30 bp from the donor and acceptor splice sites of intron 5, respectively, c.486-34delT can be defined as a deep intronic variant [63, 64]. The c.486-34delT has no effect on splicing and is predicted to be neutral. However, the clinical implications of pathogenicity of this variant are still unclear. Multiple clinical testing groups reported that it was benign, while it was observed in unrelated hemizygotes with G6PD deficiency [55–57, 65]. Recent studies have suggested that synonymous mutations are non-neutral in other genes [66–68]. Therefore, the functional effects of synonymous (c.519C>T and c.1311C>T) and intronic variants (c.486-34delT and c.1365-13T>C) should be further investigated to gain insight into the molecular mechanisms underlying G6PD deficiency. Gene expression analysis is a promising approach to better understand the effect of *G6PD* mutations. Changes in DNA and mRNA sequences can alter the rate of translation and mRNA stability, resulting in decreased protein production [69, 70]. Additionally, whole gene sequencing can be utilized to determine whether there are mutations in regions such as 5’ and 3’ UTRs and other non-coding areas which could be associated with altered gene expression and be accountable for the deficient phenotype [71].

Notably, no mutation was detected among 33 individuals who were considered as intermediate deficiency (30–80% of the AMM). Hematological factors such as white blood cell count, reticulocyte count, and age of red blood cells may affect the phenotypic test results where G6PD activity decreases significantly as erythrocytes age. Additionally, the impaired G6PD activity in these subjects may result from *G6PD* mutations in other noncoding regions, such as 5’ and 3’ UTRs, and exon 1 and intron 2, in which the designed sequencing primers cannot comprehensively detect. *G6PD* epigenetic events have been found to downregulate *G6PD* expression [72]. Alteration in other genes encoding regulatory proteins involved in the regulation of *G6PD* expression might also contribute to G6PD deficiency by decreasing transcriptional level or affecting post-translational modification of the G6PD protein [73, 74]. Because the medical history of these samples is unknown, it is possible that they have acquired G6PD deficiency. It was previously reported that reduced G6PD activity has been associated with several underlying diseases, such as diabetes and rheumatoid arthritis [75, 76]. It should also be noted that the WST-8 assay used in this study is not a standard method for measuring G6PD activity. The performance of WST-8 assay was comparable to a reference G6PD assay for identification of individuals with deficient activity (<30%), but misclassification was observed at borderline G6PD values between intermediate and normal activity [77].

To identify those who would be eligible for tafenoquine administration, phenotypic data were analyzed and a normal G6PD was defined as > 70% of the AMM. Two individuals with heterozygous *G6PD* Mahidol and *G6PD* Canton, defined as G6PD intermediate according to the 80% threshold, were classified as G6PD normal at the 70% cutoff. The enzyme activities for those with *G6PD* Mahidol and *G6PD* Canton were approximately 75% and 72% of the AMM, respectively. This result indicated that an 80% threshold, according to the WHO recommendation, is more reliable for screening G6PD deficiency in heterozygous females of the studied population. It should be noted that the use of an 80% cut-off will exclude malaria patients from effective radical treatment, leaving them vulnerable to relapse, which might result in more serious events than mild hemolysis. Nevertheless, there was no clear consensus that 70% or 80% would be a more appropriate cut-off value of G6PD enzyme activity in females with heterozygous deficiency [78].

Our findings suggest that the phenotypic test alone may be insufficient for detecting G6PD deficiency in heterozygous females. Hence, the multiplex HRM assays developed here could be used as a supplementary approach when deciding whether individuals with borderline enzyme activities should be given the drugs. Our assay can be used to identify six *G6PD* variants by one-step real-time PCR. We show that the HRM assay is a reliable method for detecting *G6PD* mutations, particularly in females with either normal or deficient G6PD activity, depending on the type of variant and zygosity. In comparison to other genotypic tests, the HRM assay offers potential advantages for rapid, simple, and high-throughput genotypic screening with up to 68 samples that can be analyzed simultaneously. Because the polymorphic frequency of the *G6PD* gene varies across different populations, our assays may be appropriate for genotyping in areas where the six *G6PD* variants are widespread but may not be useful in other regions. However, HRM assays can be further developed to detect other *G6PD* mutations, which can include G6PD variants with normal enzyme activity and rare variants that are at risk for oxidative hemolysis.

In conclusion, the developed multiplex HRM assays correctly identified the genotypes of six missense *G6PD* variants common in Thailand. Homozygous and heterozygous females could be distinguished from those with wild-type *G6PD* by monitoring changes in melting-curve shapes and *T*_*m*_. Moreover, the assays successfully detected *G6PD* mutations in heterozygous females with normal G6PD activity. The sequencing results showed a high frequency of intronic variants among all detected variants. This indicated that intronic variants may be more common than previously thought because the molecular characterization of *G6PD* has mainly focused on the protein-coding exons. We recommend comprehensive molecular analysis to detect genetic variation in the *G6PD* gene, covering both translated and untranslated regions, because some individuals with impaired enzyme activity have no identified mutations in the exon regions. Finally, the effects of synonymous and intronic variants on *G6PD* expression should be investigated to understand how these mutations cause enzyme deficiency.

## Supporting information

S1 Table The 64 *G6PD*-mutant samples used for method validation. (PDF)

S2 Table Primers used for *G6PD* amplification and sequencing. (PDF)
