# Supplementary material for: Molecular characterization of G6PD mutations identifies new mutations and a high frequency of intronic variants in Thai females
Source: PLoS One. 2023 Nov 15;18(11):e0294200. doi: 10.1371/journal.pone.0294200 (PMC10651042; doi:10.1371/journal.pone.0294200)
Supplement: S1 Table — (PDF) [file pone.0294200.s001.pdf]

S1 Table. The number of *G6PD* mutations of 64 G6PD-deficient samples used for method validation.

**Variant**	**Hemizygote**	**Heterozygote**
Gaohe (A95G)	2	1
Chinese-4 (G392T)	5	6
Mahidol (G487A)	7	6
Viangchan (G871A)	11	12
Chinese-5 (C1024T)	2	5
Union (C1360T)	6	1
**Total**	**33**	**31**
