# Supplementary material for: Molecular characterization of G6PD mutations identifies new mutations and a high frequency of intronic variants in Thai females
Source: PLoS One. 2023 Nov 15;18(11):e0294200. doi: 10.1371/journal.pone.0294200 (PMC10651042; doi:10.1371/journal.pone.0294200)
Supplement: S2 Table — (PDF) [file pone.0294200.s002.pdf]

S2 Table. Primers used for *G6PD* amplification and sequencing.

Primer name	Primer sequence (from 5' to 3')	Reference
Ex2F[a]	GGGCAATCAGGTGTCACC	[1]
Ex2R[a]	GGCTTTTAAGATTGGGGCCT	[1]
Ex3F[a]	AGACATGCTTGTGGCCCAGTA	[1]
Ex5F	GGACACTGACTTCTGAGGGCA	[1]
Ex5R[a]	AAGGGAGGGCAACGGCAA	[1]
Ex6F[a]	CACGGGGGCGAGGAGGTT	[1]
Ex8F	CGGTTTTATGATTCAGTGATA	[1]
Ex8R[a]	AGGGCATGCTCCTGGGGA	[1]
Ex9F[a]	GTGAGCAGAGCCAAGCAG	[1]
Ex11F	CAGATACAAGGTGCCCTACAG	[1]
Ex13R[a]	TGGCGGGGGTGGAGGTGG	[1]
In5F[a]	GTCTACGAGGCCGTCACC	This study
In5R[a]	CCGAAGGGCTTCTCCACGA	This study
In8F[a]	CGCCTCCACCAACTCAGATG	This study
In9R[a]	TTGCCGCAGCGCAGGATG	This study

[a] The G6PD gene was amplified using six primer sets (Ex2F−E2R, Ex3F−Ex5R, Ex6F−Ex8R, Ex9F−Ex13R, In5F−In5R, and In8F−In9R)

Reference

1. Boonyuen U, Songdej D, Tanyaratsrisakul S, Phuanukoonnon S, Chamchoy K, Praoparotai A, et al. Glucose-6-phosphate dehydrogenase mutations in malaria endemic area of Thailand by multiplexed high-resolution melting curve analysis. Malar J. 2021;20(1):194. doi: 10.1186/s12936-021-03731-0.
